# Continuous glucose monitoring demonstrates low risk of clinically significant hypoglycemia associated with sulphonylurea treatment in an African type 2 diabetes population: results from the OPTIMAL observational multicenter study

**DOI:** 10.1136/bmjdrc-2021-002714

**Published:** 2022-04-21

**Authors:** Anxious J Niwaha, Lauren R Rodgers, Alice L J Carr, Priscilla A Balungi, Raymond Mwebaze, Andrew T Hattersley, Beverley M Shields, Moffat J Nyirenda, Angus G Jones

**Affiliations:** 1 Institute of Biomedical and Clinical Science, College of Medicine and Health, University of Exeter Medical School, Exeter, UK; 2 NCD Theme, MRC/UVRI and LSHTM Uganda Research Unit, Entebbe, Uganda; 3 Institute of Health Research, University of Exeter Medical School, Exeter, UK; 4 Department of Medicine, St. Francis Hospital Nsambya, Kampala, Uganda; 5 Macleod Diabetes and Endocrine Centre, Royal Devon and Exeter NHS Foundation Trust, Exeter, UK; 6 NCD Epidemiology, London School of Hygiene & Tropical Medicine, London, UK

**Keywords:** CGM, Developing Countries, Diabetes Mellitus, Type 2, Hypoglycemia

## Abstract

**Introduction:**

People living with diabetes in low-resource settings may be at increased hypoglycemia risk due to food insecurity and limited access to glucose monitoring. We aimed to assess hypoglycemia risk associated with sulphonylurea (SU) and insulin therapy in people living with type 2 diabetes in a low-resource sub-Saharan African setting.

**Research design and methods:**

This study was conducted in the outpatients’ diabetes clinics of two hospitals (one rural and one urban) in Uganda. We used blinded continuous glucose monitoring (CGM) and self-report to compare hypoglycemia rates and duration in 179 type 2 diabetes patients treated with sulphonylureas (n=100) and insulin (n=51) in comparison with those treated with metformin only (n=28). CGM-assessed hypoglycemia was defined as minutes per week below 3mmol/L (54mg/dL) and number of hypoglycemic events below 3.0 mmol/L (54 mg/dL) for at least 15 minutes.

**Results:**

CGM recorded hypoglycemia was infrequent in SU-treated participants and did not differ from metformin: median minutes/week of glucose <3 mmol/L were 39.2, 17.0 and 127.5 for metformin, sulphonylurea and insulin, respectively (metformin vs sulphonylurea, p=0.6). Hypoglycemia risk was strongly related to glycated haemoglobin (HbA1c) and fasting glucose, with most episodes occurring in those with tight glycemic control. After adjusting for HbA1c, time <3 mmol/L was 2.1 (95% CI 0.9 to 4.7) and 5.5 (95% CI 2.4 to 12.6) times greater with sulphonylurea and insulin, respectively, than metformin alone.

**Conclusions:**

In a low-resource sub-Saharan African setting, hypoglycemia is infrequent among people with type 2 diabetes receiving sulphonylurea treatment, and the modest excess occurs predominantly in those with tight glycemic control.

**Significance of this study**

**What is already known about this subject?**

- Evidence from high-income countries suggest that severe hypoglycemia is rare in patients taking sulphonylureas, but in those with well-controlled diabetes, non-severe hypoglycemia may be common.
- People treated with sulphonylureas in low-income countries may be at increased of hypoglycemia because of food insecurity, lack of access to glucose monitoring, and use of older sulphonylurea agents that have higher hypoglycemia risk; however, the risk of hypoglycemia with these agents in low-income populations is unclear.

**What are the new findings?**

- Both continuous glucose monitoring assessed and self-reported hypoglycemia were infrequent in participants with sulphonylurea-treated diabetes and did not differ from metformin.
- Hypoglycemia risk was strongly associated with glycemic control, with most episodes occurring in those with tight glycemic control.
- After adjusting for glycemic control (HbA1c), participants receiving sulphonylurea or insulin treatment experienced two and five times more continuous glucose monitoring assessed hypoglycemia, respectively, than those receiving metformin.

**How might these results change the focus of research or clinical practice?**

- The high rates of poor glycemic control in type 2 diabetes patients and relatively low hypoglycemic events among patients taking sulphonylureas suggest that there is room for optimizing glycemic control using these cheap, readily available and effective agents in low-resource settings.

## Introduction

The prevalence of type 2 diabetes is rapidly increasing especially in low-income and middle-income countries (LMICs) where the majority of people living with type 2 diabetes reside.^1^ While complications of type 2 diabetes can be reduced by maintaining glucose control,^2 3^ glycemic control for people living with type 2 diabetes in LMICs is often poor.^4^ A key barrier to intensifying glucose-lowering therapy in low-resource healthcare settings is fear of hypoglycemia.^5 6^ Sulphonylureas (SUs) and insulin remain the most available treatments after metformin for people living with diabetes in LMICs.^7 8^ Because of limited resources, treatments with lower risk of hypoglycemia, such as the newer classes of SUs (eg, gliclazide and glimepiride) and analog insulins, are not readily available in LMICs,^8^ and robust glucose monitoring is often unaffordable, even for those treated with insulin.^9^ Concerns about hypoglycemia mean that SUs may be started at far higher glycemic thresholds than recommended in international guidance.^10 11^

It is not clear whether this fear of hypoglycemia among type 2 diabetes patients in low-resource settings is justified. Previous studies investigating the burden of hypoglycemia among type 2 diabetes patients in low-resource settings are limited, with available data predominantly from high-income countries.^12^ Observational and trial data from high-income countries suggest that severe hypoglycemia is rare in patients taking SUs, but in those with well-controlled diabetes, non-severe hypoglycemia may be common.^13 14^ Studies in high-income countries suggest substantially higher rates of hypoglycemia with insulin than SUs.^15 16^ However, these data may not apply in resource poor settings where use of older SUs, with higher hypoglycemia risk compared with newer generation SUs (eg, gliclazide and glimepiride) and food insecurity (and therefore missed meals) are common. In addition, due to resource constraints, the majority of those receiving treatment associated with hypoglycemia will not be able to access capillary glucose monitoring.

We therefore aimed to assess hypoglycemia risk with SUs and insulin therapy (in comparison with metformin) in people living with type 2 diabetes in a low-resource sub-Saharan African setting.

## Methods

We compared continuous glucose monitoring (CGM) and self-reported hypoglycemia in people treated with metformin, sulfonylureas or insulin attending diabetes clinics in Uganda. CGM was used to obtain an objective assessment of hypoglycemia.

### Study population

People living with type 2 diabetes attending a routinely scheduled diabetes clinic in a rural-based hospital (Masaka regional referral hospital) and urban-based hospital (St. Francis Hospital Nsambya) were invited consecutively. Eligible individuals were aged 18 years and above and treated with metformin, SU or insulin. All participants provided written informed consent before entering the study.

### Patient and public involvement (PPI)

Patients were involved in prioritization of the research question. Patients were not involved in the design and conduct of the study. However, they were central to dissemination of the results by choosing to have some of the results sent to their respective clinicians and will continue to be involved in ongoing study dissemination.

### Study procedures

We used questionnaires to record baseline patient characteristics including sociodemographic, diabetes medical history, current treatment information, and history of severe hypoglycemia in the previous 12 months.

We assessed glucose levels over a 14-day period from the baseline visit using the blinded Freestyle Libre Pro Glucose Monitoring System (Abbott Laboratories, Illinois, USA) as previously described.^17^

### Hypoglycemia assessment

CGM-assessed hypoglycemia was defined according to the international consensus on use of CGM guidelines as the number of hypoglycemic events that occur over the given CGM reporting period.^18^ Clinically significant hypoglycemic events were defined as readings below the 3.0 mmol/L (54 mg/dL) threshold for at least 15 minutes. The end of a CGM hypoglycemic event was defined at the point where glucose was at least 3.9 mmol/L (70 mg/dL) for 15 min. Hypoglycemia rate and duration below 3 mmol/mol were standardized to events/week and minutes/week per week, respectively, to account for variation in duration of CGM measurement. Self-reported hypoglycemia data were collected using a questionnaire that captured the history of hypoglycemia requiring assistance of another person, history and number of times the participant was hospitalized due to hypoglycemia in the previous 12 months.

### Statistical analysis

Statistical analysis was performed using Stata V.16.1 (StataCorp LLC).

Medians and IQrs are reported for descriptive data due to skewed nature of most variables. We compared median hypoglycemia event rate per week and the median minutes below 3 mmol/L per week across treatment classes using the non-parametric Wilcoxon rank-sum test. Frequency of self-reported hypoglycemia and hospital admission due to hypoglycemia was assessed, and proportions were compared across the three treatment groups using χ^2^ or Fischer’s exact tests.

Hypoglycemia rate and minutes below 3 mmol/L per week results were positively skewed following a Poisson distribution. We therefore assessed whether the differences in hypoglycemia rates between the three treatment groups were due to confounding by differences in clinical features associated with hypoglycemia using Poisson regression models. To ensure model assumptions of variance, we fitted Poisson regression with robust SEs.^19^ The differences in minutes below 3 mmol/L were also assessed using Poisson regression; the Poisson regression with robust SEs (Huber-White-Sandwich linearized estimator of variance) was preferred to log-linear regressions for easy interpretation of results and due to the presence of numerous natural zeros in the outcome of interest (minutes below 3 mmol/L) and overdispersion.^20^ We assessed the rates and the minutes below 3 mmol/L, with and without adjustment for glycemic control (glycated haemoglobin (HbA1c) or fasting plasma glucose (FPG)), age, sex, diabetes duration and body mass index (BMI). We then visually assessed the relationship between FPG and HbA1c using scatter plots and compared rate and duration at different HbA1c and FPG values.

The adjusted means of hypoglycemia rates and minutes below 3 mmol/L per week were then estimated using the margins command for each treatment class (ie, metformin only, SUs and insulin) holding HbA1c or FPG (or other adjusted covariates) at the sample population mean. We also estimated adjusted mean rates of hypoglycemia and minutes per week below glucose levels of 3 mmol/L at clinically relevant HbA1c and FPG thresholds.

## Results

### Baseline characteristics

One hundred and seventy-nine participants met analysis inclusion criteria (online supplemental figure 1). Twenty-eight participants were treated with metformin only, 100 were treated with SUs (with or without metformin) and 51 were treated with insulin (with or without metformin and/or SU) (online supplemental figure 1). Of the 100 participants treated with SUs, 67 patients (67%) were prescribed glibenclamide, 26 (26%) were prescribed glimepiride and 7 (7%) were prescribed gliclazide. Forty-two of 51 (78.8%) of the patients taking insulin were on mixtard insulin. The median duration of CGM was 14 (IQR: 13–14) days. Baseline characteristics are shown in table 1. Participants treated with SU and insulin had substantially higher glycemia than those treaded with metformin: median HbA1c (mmol/mol) of 66 (IQR: 2–83), 84 (IQR: 67–102) and 46 (IQR: 39.5–63.5) respectively.

10.1136/bmjdrc-2021-002714.supp1 Supplementary data

**Table 1:** Characteristics of CGM-assessed and self-reported hypoglycemia in type 2 diabetes according to treatment

Variable	Median (IQR) for continuous variables, n (%) for proportions		
	Metformin group	SU group	Insulin group
Number	28	100	51
Female, n (%)	18 (64.3)	57 (57.0)	31 (60.8)
Age, years	56.5 (49.5–61.5)	55.5 (50.0–62.0)	55.0 (49.0–64.0)
Diabetes duration, years	5.0 (2.0–8.0)	6.0 (3.0–9.0)	10.0 (8.0–17.0)
BMI, kg/m^2^	26.9 (24.2–29.9)	26.7 (23.7–30.1)	25.8 (23.1–30.2)
eGFR	113.4 (96.8–123.7)	112.8 (93.8–121.0)	110.8 (92.3–121.8)
Renal impairment, n (%)	0 (0)	6 (6.0)	4 (7.8)
**Glycemic control**			
CGM duration	14 (13–14)	14 (13–14)	14 (13–14)
Average CGM glucose (mmol/L)	6.8 (5.4–9.9)	8.5 (7.0–12.0)	10.1 (8.2–14.5)
HbA1c (%)	6.4 (5.8–8.0)	8.2 (6.9–9.6)	9.8 (8.2–11.3)
HbA1c (mmol/mol)	46 (40–64)	66 (52–83)	84 (67–102)
Fasting glucose	7.2 (5.5–10.2)	8.2 (6.2–10.7)	9.3 (7.0–12.3)
Glucose variability (cv)	0.29 (0.26–0.33)	0.34 (0.29–0.39)	0.39 (0.33–0.47)
SD	2.06 (1.65–2.93)	3.16 (2.59–3.85)	4.0 (3.3–5.2)
Percent time spent in optimal range	78.1 (55.3–86.4)	60.1 (33.8–73.9)	40.1 (22.2,–55.4)
Percent time above 10	10.9 (1.3–35.3)	31.9 (14.3–66.0)	49.3 (30.8–74.2)
**CGM hypoglycemia per week**			
Episodes <3 mmol/L	1 (0–2.3)	0.5 (0–3.0)	2 (0–6.0)
Total time/week <3 mmol/L, min	39.2 (0–174.8)	17.0 (0–229.3)	127.5 (0–637.5)
**Per cent time <3 mmol/L (%)**	0.39 (0, 1.74)	0.17 (0, 2.26)	1.27 (0, 6.42)
**Self-reported hypoglycemia, n (%)**			
History of hypoglycemia events, n (%)	7 (25.0)	28 (28.0)	23 (45.1)
Hospitalized for hypoglycemia in the previous 12 months, yes	1 (3.6)	3 (3.0)	6 (11.8)
Hospitalized for hypoglycemia in the previous 12 months, % (95% CI)	3.6 (0.1 to 18.3)	3.0 (0.6 to 8.5)	11.8 (4.4 to 23.9)

BMI, body mass index; CGM, continuous glucose monitoring; eGFR, estimated glomerular filtration rate; HbA1c, glycated haemoglobin; SU, sulphonylurea.

Metformin group includes patients being treated with metformin only, SU group includes patients on SUs and metformin, and insulin group includes patients being treated with insulin with metformin and/or SUs. Renal impairment was defined as an estimated glomerular filtration rate (eGFR)<60 mL/min/1.73 m^2^. Per cent time spent in optimal range was defined as the percentage of readings and time spent between 3.9–10.0 mmol/L (70–180 mg/dL).

### Hypoglycemia was infrequent in participants with SU-treated diabetes and did not differ from metformin

**Figure 1:**
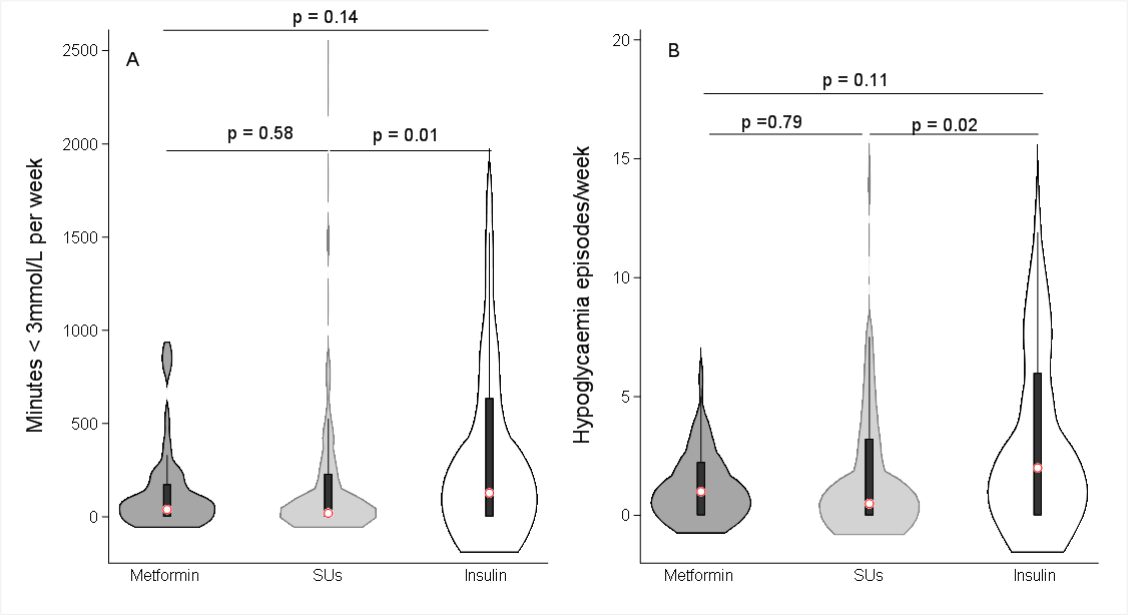
The distributions of hypoglycemia measured by CGM in individuals treated with metformin only, or sulphonylureas (SU) (with or without metformin) and insulin (with or without metformin and/or sulfonylureas). CGM, continuous glucose monitoring.

Median minutes and rate below 3 mmol/L per week of CGM defined hypoglycemia were low in those treated with SUs and similar to rates observed in those treated with metformin (figure 1 and table 1). Median (IQR) minutes below 3 mmol/L per week were 39.2 (0–174.8), 17.0 (0–229.3) and 127.5 (0–637.5) with metformin, SU, and insulin, respectively. Median hypoglycemic events/week were 1 (IQR: 0–2.3), 0.5 (0–3.0) and 2 (0–6.0) with metformin, SU, and insulin, respectively. Self-reported hypoglycemia results were broadly consistent with CGM findings, with numerically similar proportions of reported hypoglycemia-related hospitalization with SU (3.0% (95% CI 0.6 to 8.5) and metformin (3.6% (95% CI 0.1 to 18.3)) and higher rates in those treated with insulin (11.8% (95% CI 4.4 to 23.9) (table 1).

### Hypoglycemia risk was strongly associated with glycemic control, with most episodes occurring in tightly controlled diabetes

**Figure 2:**
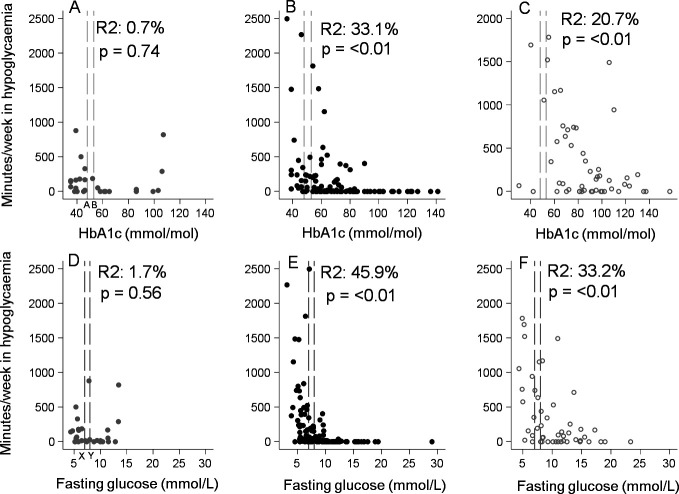
Comparison of glycemic control and hypoglycemia duration (minutes per week <3 mmol/L). Graphs in the top row show the relationship between HbA1c and the number of minutes spent in hypoglycemia per week for metformin (A), sulphonylureas (B), and insulin (C) treated participants, respectively. The bottom row shows the relationship between fasting glucose and number of minutes spent in hypoglycemia per week for metformin (D), sulphonylurea (E) and insulin (F) treated participants, respectively. The long-dashed lines denote glycemic thresholds, HbA1c 6.5% (48 mmol/mol) and 7.0% (53 mmol/mol) (top row), fasting glucose 7.0 mmol/L and 8.0 mmol/L (bottom row). HbA1c, glycated haemoglobin.

In those treated with SU and insulin, time spent in hypoglycemia and hypoglycemic event rate was strongly associated with glycemic control, with differences in HbA1c explaining 33.1% (p=<0.001) and 20.7% (p=0.005) of variation in time below 3 mmol/L for SU and insulin, respectively (figure 2). The majority of hypoglycemia occurred in those with lower HbA1c or fasting glucose (figure 2 (time <3 mmol/L) and online supplemental figure 2) (hypoglycemia rate). Participants with HbA1c below 53 mmol/mol (7%) spent 2.34% (IQR: 0.60–4.49) and 5.61% (0.34–13.80) of their total time per week in hypoglycemia (<3 mmol/L), for SU and insulin, respectively. In comparison, those who had an HbA1c ≥53 mmol/mol on SU spent 0.0% (IQR: 0.00–0.92) and those on insulin spent 1.27% (0.00–5.75) of their total time per week in hypoglycemia (<3 mmol/L). Participants with fasting glucose <7 mmol/L spent 2.40% (IQR: 0.60–4.98) and 6.52% (IQR: 1.24– 13.50) of their total time per week in hypoglycemia, for SU and insulin, respectively, in comparison with only 0.0% (IQR: 0.00–0.46) and 0.67% (IQR: 0.00–3.44) for those who had fasting glucose ≥7 mmol/L (online supplemental table 1).

### In analysis adjusted for HbA1c participants receiving SU or insulin treatment experienced two and five times more hypoglycemia, respectively, than those receiving metformin

Table 2 shows mean and rate ratio for minutes in hypoglycemia by treatment (relative to metformin), unadjusted and with adjustment for HbA1c (model 2) and HbA1c, age, diabetes duration, BMI and sex (model 3). In unadjusted analysis, the mean number of minutes <3 mmol/L per week for SU and metformin treatment did not substantially differ (duration ratio SU vs metformin 1.4 (95% CI 0.69 to 2.91), p=0.35), but duration in hypoglycemia substantially higher with insulin than metformin (duration ratio 2.5 (95% CI 1.3 to 5.0), p=0.009). After adjusting for HbA1c, differences between therapies were accentuated, with minutes <3 mmol/mol 2.1 (95% CI 0.9 to 4.7, p value=0.067) and 5.5 (95% CI 2.4 to 12.6, p value=<0.001) times greater than metformin with SU and insulin, respectively. Findings were not substantially altered by further adjustment for age, BMI, diabetes duration, renal impairment and sex.

**Table 2:** Number of minutes <3 mmol/L per week in type 2 diabetes patients on different glucose-lowering agents before and after adjusting for HbA1c and clinical features

	Variables	Minutes <3 mmol/L (95% CI)	Duration ratio (vs metformin)	P value
Model 1 R^2^=0.05	Metformin (Ref)	146.0 (60.6 to 231.3)	1.0	
	SU	206.7 (119.2 to 294.2)	1.4 (0.7 to 2.9)	0.345
	Insulin	365.9 (229.9 to 501.9)	2.5 (1.3 to 5.0)	**0.009**
Model 2 R^2^=0.23	Metformin	74.0 (14.6 to 133.4)	1.0	
	SU	156.9 (97.6 to 216.3)	2.1 (0.9 to 4.7)	0.067
	Insulin	405.7 (262.1 to 549.3)	5.5 (2.4 to 12.6)	**<0.001**
Model 3	Metformin	96.4 (20.2 to 172.6)	1.0	
R^2^=0.30	SU	157.5 (97.6 to 217.4)	1.6 (0.7 to 3.6)	0.230
	Insulin	355.0 (212.7 to 497.2)	3.7 (1.5 to 9.3)	**0.006**

Model 1: unadjusted; model 2: adjusted for HbA1c; model 3: adjusted for HbA1c, age, diabetes duration, BMI, sex, and renal impairment. Adjusted minutes <3 mmol/L are adjusted to the mean value for the covariate for the cohort (mean cohort HbA1c 73.2 mmol/mol). 95% CIs are shown in the parentheses. Renal impairment was defined as an estimated glomerular filtration rate <60 mL/min/1.73 m^2^.

Values shown are mean (95 % CIs) and p-value. Bold values denote statistical significance at the p < 0.005 level.

BMI, body mass index; HbA1c, glycated haemoglobin; SU, sulphonylurea.

When adjusting to HbA1c of 53 mmol/mol (7%), an internationally recognized target for glycemic control, estimated minutes in hypoglycemia (per week) were 137.2 (95% CI 49.6 to 224.7), 290.9 (168.8 to 413.0) and 751.9 (433.9 to 1070.0) with metformin, SU and insulin, respectively (online supplemental material 3). Findings were similar for hypoglycemia rates per week, with rates approximately two and five times higher with SU and insulin than metformin after adjustment for HbA1c (table 3). Estimated adjusted mean rates of hypoglycemia at a range of clinically relevant HbA1c (and FPG) thresholds are shown in online supplemental figure 4).

**Table 3:** Hypoglycemia rates in type 2 diabetes patients on different glucose-lowering agents before and after adjusting for HbA1c and clinical features

	Variables	Rates (95% CI)	Rate ratio (vs metformin)	P value (verses metformin)
Model 1 R^2^=0.03	Metformin (reference)	1.3 (0.7 to 1.9)	1.0	
	SUs	2.1 (1.4 to 2.8)	1.6 (0.9 to 2.7)	0.108
	Insulin	3.2 (2.1 to 4.2)	2.4 (1.4 to 4.2)	**0.002**
Model 2 R^2^=0.21	Metformin (reference)	0.6 (0.3 to 1.0)	1.0	
	SUs	1.5 (1.1 to 2.0)	2.4 (1.4 to 4.1)	**0.001**
	Insulin	3.8 (2.3 to 4.6)	5.4 (3.0 to 9.9)	**<0.001**
Model 3 R^2^=0.24	Metformin (reference)	0.7 (0.3 to 1.1)	1.0	
	SUs	1.6 (1.1 to 2.0)	2.1 (1.2 to 3.6)	**0.006**
	Insulin	3.2 (2.0 to 4.4)	4.4 (2.2 to 8.7)	**<0.001**

Model 1: unadjusted; model 2: adjusted for HbA1c; model 3: adjusted for HbA1c, age, diabetes duration, BMI, sex and renal impairment. Adjusted rates are adjusted to the mean value for the covariate for the cohort (mean cohort HbA1c 73 mmol/mol). Renal impairment was defined as an estimated glomerular filtration rate <60 mL/min/1.73 m^2^.

Values shown are mean (95% CIs) and p-value. Bold values denote statistical significance at the p < 0.05 level.

BMI, body mass index; HbA1c, glycated haemoglobin; SUs, sulphonylureas.

## Discussion

This study has demonstrated that both CGM assessed and self-reported clinically significant hypoglycemia in participants treated with SUs in Uganda is infrequent among patients who receive SU treatment. While observed hypoglycemia rates and duration were similar in those treated with metformin and SU, hypoglycemia risk was strongly associated with glycemic control, and after adjusting for differences in HbA1c, the risk of hypoglycemia doubled and quintupled in those treated with SUs and insulin, respectively. The modest hypoglycemia excess associated with SUs in comparison with metformin occurred predominantly in those with tight glycemic control. Hypoglycemia was more common in insulin treated diabetes than those treated with SU, further increasing on adjustment for glycemic control.

Studies comparing hypoglycemia risk across different treatments in type 2 diabetes are limited in LMICs, especially sub-Saharan Africa. The few hypoglycemia-related studies among people with type 2 diabetes patients in sub-Saharan Africa that have assessed the incidence and prevalence of hypoglycemia have predominantly used self-reported hypoglycemia and documented increased risk with insulin use.^21^ The majority of these studies either included only patients on insulin and or grouped SUs together with other oral glucose-lowering agents.^11 21 22^ Our finding that SU treatment is associated with a modest risk of clinically significant hypoglycemia among those with type 2 diabetes is consistent with studies in other popualtions.^23 24^ However, it should be noted that the SUs in these studies are of newer generation, like gliclazide and glimepiride, that are known to have a lower hypoglycemia risk compared with glibenclamide.^7^ The present study, although not designed to compare intra-SU class differences, showed a modest hypoglycemia risk even when majority (two out of three) of our patient population were taking glibenclamide, an older agent with higher hypoglycemia risk.^7^ Moreover, the modest hypoglycemia excess in the SUs group mainly occurred in a small proportion of patients with tightly controlled diabetes, below international glycemic targets.^25–27^

A key strength of this study is the objective assessment of hypoglycemia through use of blind CGM monitoring. This removed potential biases that could arise from patient reactivity to glucose measurements, differences in glucose testing by treatment, hypoglycemia unawareness and recall bias that may affect studies assessing self-reported hypoglycemia or using medical records. An additional strength is comparison across therapies. It is well known that CGM can report occurrence of hypoglycemia in those who do not have diabetes, or are treated with medications not associated with hypoglycemia risk,^28 29^ meaning the absolute risk of meaningful hypoglycemia by CGM will be overestimated. By including a metformin ‘control’ arm in our study, we ensured to avoid this overestimation by assessing the excess risk. A notable limitation of our study was that routine capillary glucose monitoring is not available to the vast majority of people with diabetes in Uganda, due to cost. Therefore, self-reported hypoglycemia is very unlikely to have been confirmed by glucose testing and is likely to be inaccurate in a population like ours where healthy literacy including hypoglycemia education is not good. Such testing may even be limited in a healthcare setting. Additionally, the modest number of participants treated with only metformin will have impacted our ability to detect modest differences in hypoglycemia risk in comparisons against metformin, as shown by the large CIs of estimates for metformin treated participants. Lastly, the majority of participants with SU and insulin treated diabetes had poor glycemic control, while this reflects current practice in this region, given the strong relationship between glycemic control and hypoglycemia risk, it is likely that hypoglycemia rates would be substantially higher were glycemic control improved in this population, as suggested by our adjusted analysis.

Glycemic control is the cornerstone of lowering microvascular complications among people living with diabetes. While there is no doubt that there is an association between SUs (especially the older agents like glibenclamide) and insulin treatment and hypoglycemia, the high rates of poor glycemic control in type 2 diabetes patients and relatively low hypoglycemic events among patients taking SUs suggest that there is room for optimizing glycemic control using these cheap, readily available and effective agents, despite the specific challenges of food insecurity and lack of glycemic monitoring in many LMIC populations. This supports the recommendations to optimize glycemic control using these readily available and affordable agents including metformin and SUs.^8 30^ The modest excess of hypoglycemia was predominantly seen in a small proportion of patients taking SUs whose fasting glucose was less than 7 mmol/L or HbA1c <7% (53mmol/mol) (thresholds often recommended by international guidelines) suggesting caution is needed when treating below these levels.^27^

In conclusion in a low resource sub-Saharan African setting, clinically significant hypoglycemia is infrequent among people with type 2 diabetes receiving SU treatment, and the modest excess occurs predominantly in those with tight glycemic control.

## Data Availability

Data are available on reasonable request. Data analyzed in this study are available to researchers on reasonable request from the corresponding author.
