# Supplementary material for: Continuous glucose monitoring demonstrates low risk of clinically significant hypoglycemia associated with sulphonylurea treatment in an African type 2 diabetes population: results from the OPTIMAL observational multicenter study
Source: BMJ Open Diabetes Res Care. 2022 Apr 21;10(2):e002714. doi: 10.1136/bmjdrc-2021-002714 (PMC9024213; doi:10.1136/bmjdrc-2021-002714)
Supplement: Supplementary data [file bmjdrc-2021-002714supp001.pdf]

BMJ Publishing Group Limited (BMJ) disclaims all liability and responsibility arising from any reliance placed on this supplemental material which has been supplied by the author(s)

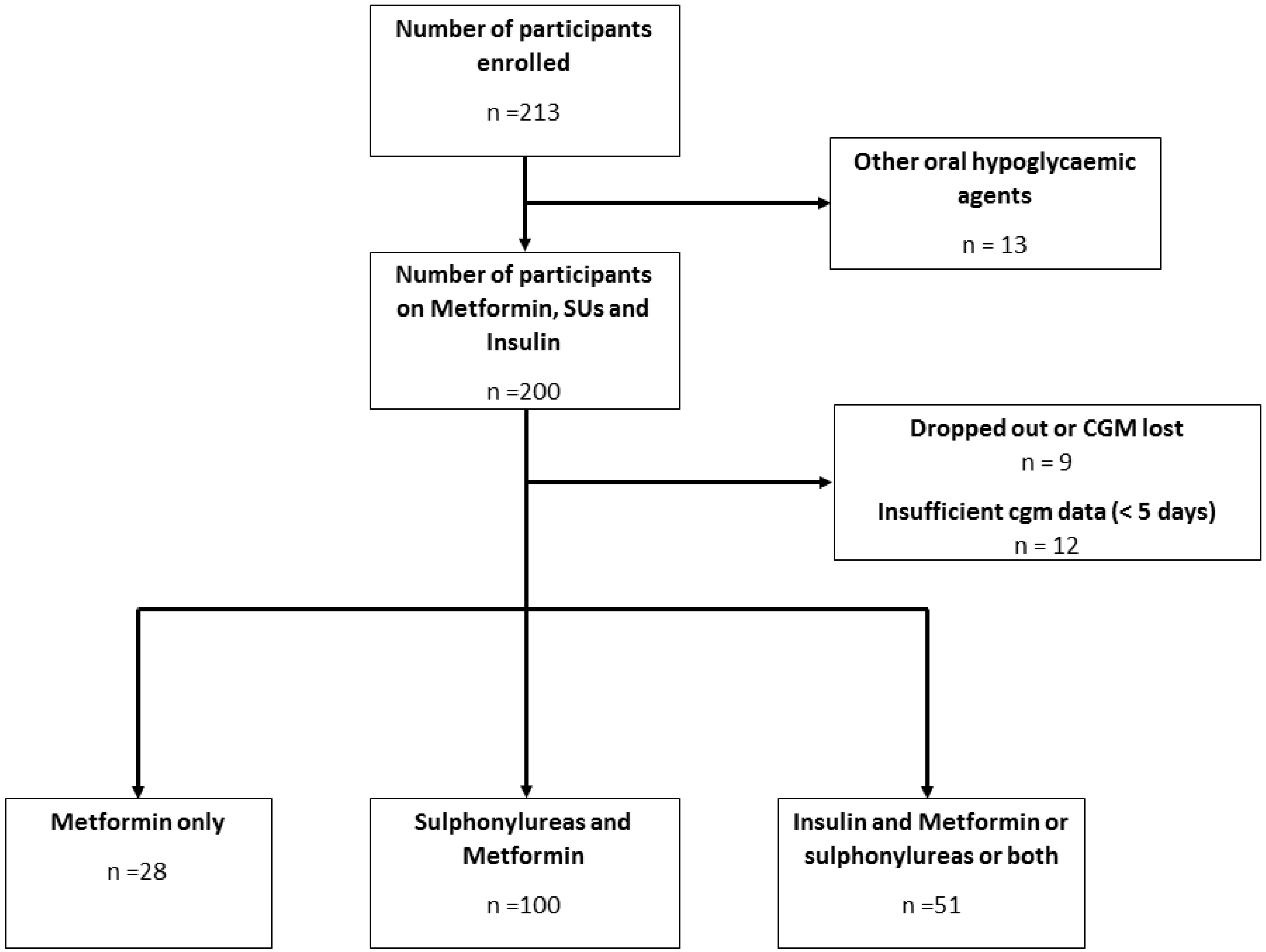

Supplementary figure 1: Participant flow chart

Supplementary Table 1: Percentage of time below 3 mmol/L per week stratified by type of medication and glycaemia control

	Metformin		SUs		Insulin	
**HbA1c (mmol/mol)**	HbA1c < 53	HbA1c ≥ 53	HbA1c < 53	HbA1c ≥ 53	HbA1c < 53	HbA1c ≥ 7.0 %
Percentage time < 3 mmol/L per week	1.42 (0.15, 1.8)	0.00 (0.00, 0.52)	2.34 (0.60, 4.49)	0.00 (0.00, 0.92)	5.61 (0.34, 13.80)	1.27 (0.00, 5.75)
**FPG (mmol/L)**	FPG < 7.0	FPG ≥ 7.0	FPG < 7.0	FPG ≥ 7.0	FPG < 7.0	FPG ≥ 7.0
Percentage time < 3 mmol/L per week	1.42 (0.15, 1.8)	0.15 (0.00, 1.68)	2.40 (0.60, 4.98)	0.00 (0.00, 0.46)	6.52 (1.24, 13.50)	0.67 (0.00, 3.44)

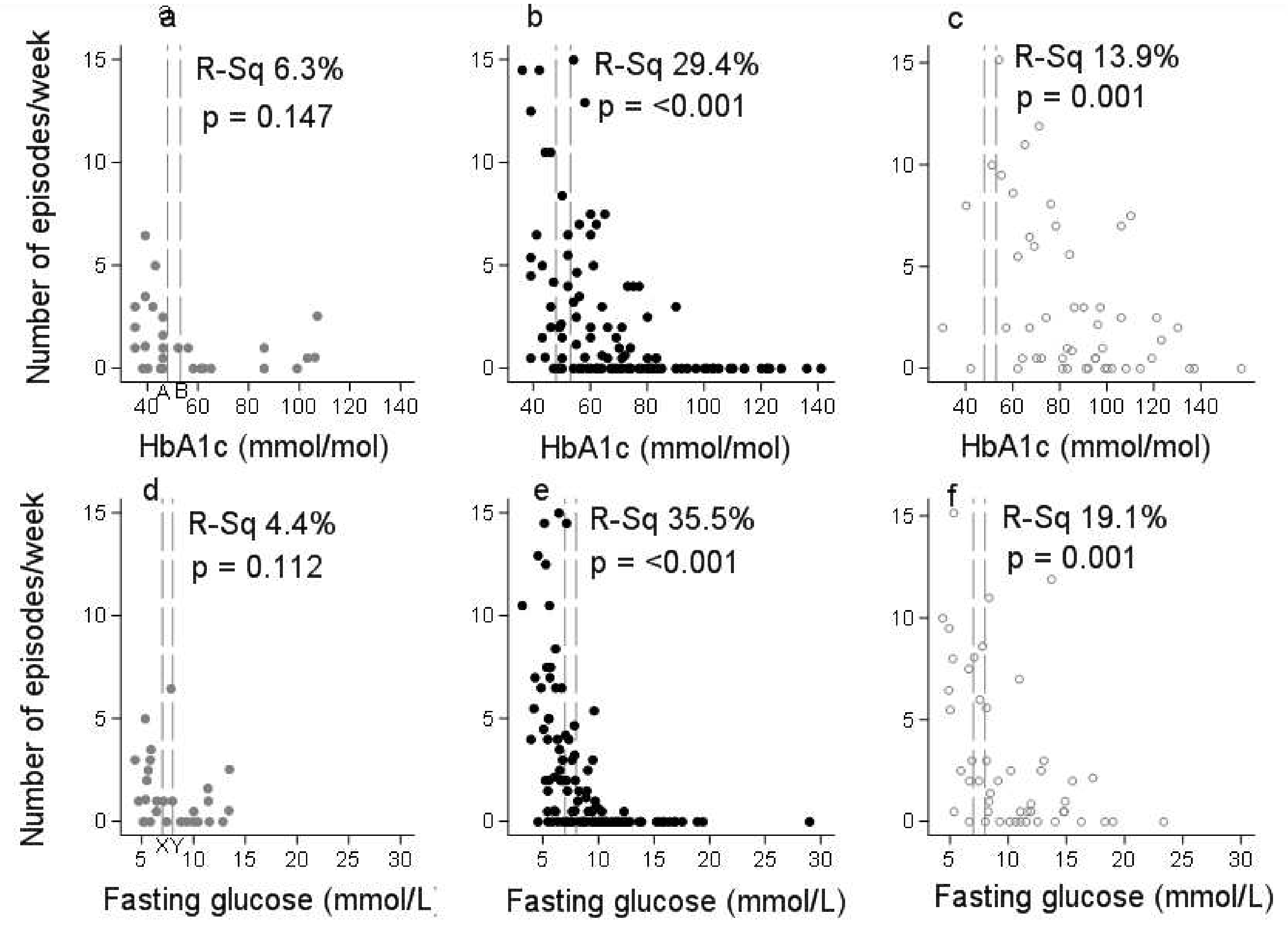

Supplementary figure 2: Comparison of HbA1cand Hypoglycaemia rates and duration per week.

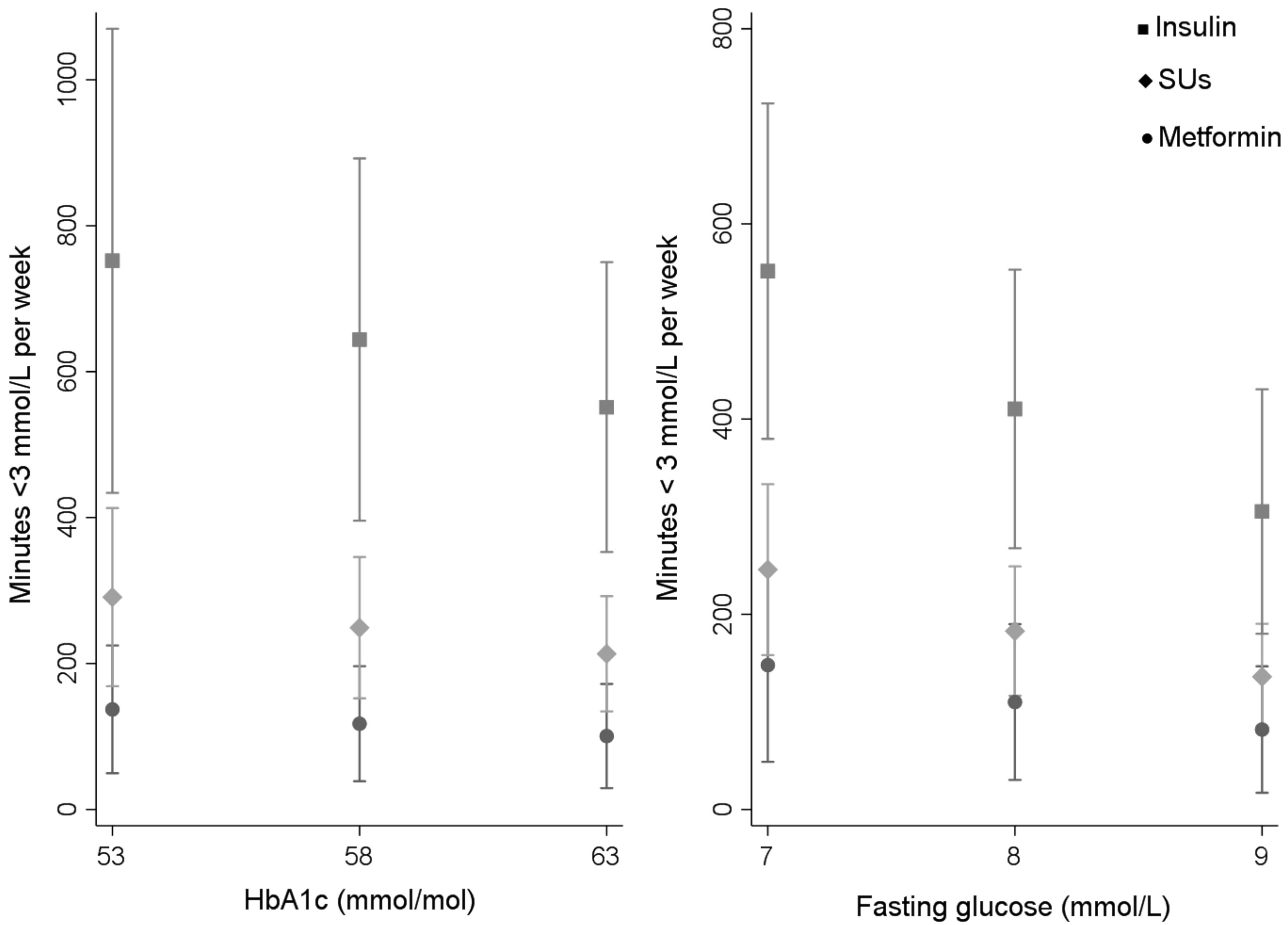

Supplementary figure 3: Estimated number of minutes below 3 mmol/L per week at different HbA1c and fasting plasma glucose levels. The error bars denote 95% confidence intervals.

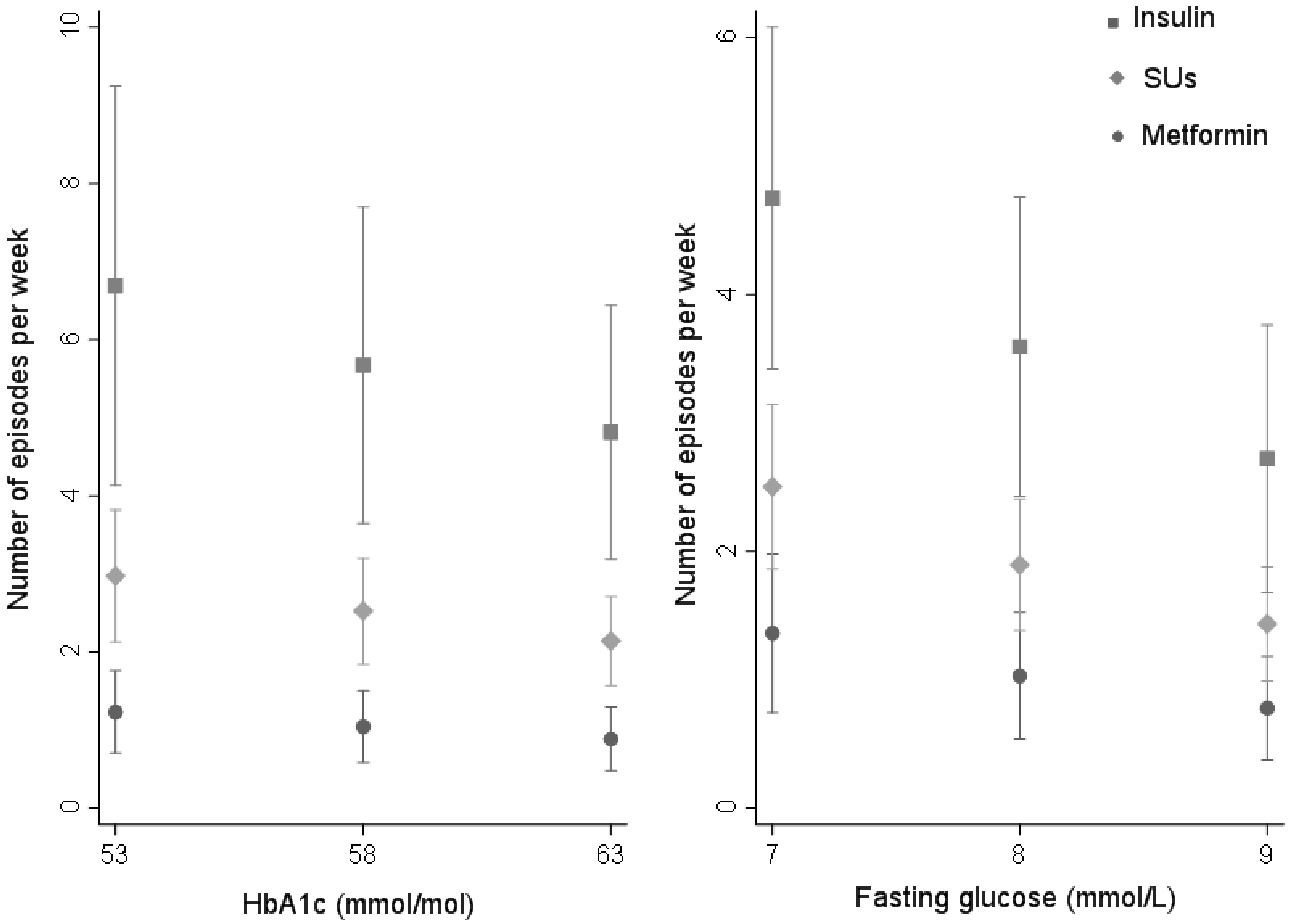

Supplementary figure 4: Estimated number of minutes below 3 mmol/L per week at different HbA1c and fasting plasma glucose levels. The error bars denote 95% confidence intervals.
